# Long-term trends in the burden of edentulism in China over three decades: A Joinpoint regression and age-period-cohort analysis based on the global burden of disease study 2019

**DOI:** 10.3389/fpubh.2023.1099194

**Published:** 2023-04-27

**Authors:** Xiaofeng Qin, Jinan He, Haoyu He, Xihua Yuan, Xiaohui Su, Xiaojuan Zeng

**Affiliations:** ^1^Guangxi Key Laboratory of Oral and Maxillofacial Rehabilitation and Reconstruction, College of Stomatology, Hospital of Stomatology, Guangxi Medical University, Nanning, China; ^2^Department of Dermatology and Venerology, The Second Affiliated Hospital, Guangxi Medical University, Nanning, China; ^3^Guangxi Health Commission Key Laboratory of Prevention and Treatment for Oral Infectious Diseases, College of Stomatology, Hospital of Stomatology, Guangxi Medical University, Nanning, China; ^4^Department of Oral Health Policy Research, College of Stomatology, Hospital of Stomatology, Guangxi Medical University, Nanning, China

**Keywords:** age-period-cohort analysis, years lived with disability, incidence, prevalence, edentulism

## Abstract

**Background:**

To investigate secular trends in edentulism incidence, prevalence, and years lived with disability (YLDs) rates in Chinese men and women from 1990 to 2019.

**Methods:**

Data were obtained from the Global Burden of Disease Study 2019. The annual percentage change and average annual percentage change were calculated using Joinpoint regression analysis. The age-period-cohort (APC) analysis estimated the independent age, period, and cohort effects.

**Results:**

From 1990 to 2019, the crude incidence, prevalence, and YLDs of edentulism in the Chinese population increased year by year, while the age-standardized incidence, prevalence, and YLDs decreased, and the latter was higher in women than in men. The APC analysis showed that the age effect increased in men and women from age 20 to 74 and decreased thereafter. The risk of tooth loss increased with age. However, the relationship was not linear. The temporal effect showed a gradual increase; the risk of missing teeth gradually increased with the changing modern living environment. The cohort effect showed a single decreasing trend, with the early birth cohort having a higher risk of tooth loss than the later birth cohort population. The age, period, and cohort effects were consistent for both sexes.

**Conclusion:**

Although the standardized incidence, prevalence, and YLD rate and cohort effect of dentition loss in China are declining, they are still causing a severe burden to China due to the continued aging of the population and the rising period effect. Despite the decreasing trends of the standardized incidence and prevalence of dentition loss and the rate of YLDs, China should develop more effective oral disease prevention and control strategies to reduce the increasing burden of edentulism in the older adult, especially in older women.

## 1. Introduction

Edentulism is the absence of all natural teeth in the mouth, also known as edentulous jaw. The edentulous jaw can affect people’s physical and mental health by reducing chewing ability, which can lead to a lack of food choices, thus altering nutritional intake [1]. Additionally, impaired masticatory function results in decreased tongue pressure, swallowing function, maximum bite force and oral masticatory motor function, leading to cognitive decline in the older adult [2–4]. Additionally, edentulous jaws can lead to loss of alveolar bone around the teeth [5], which can alter facial appearance and articulation clarity, thereby affecting patients’ mental health and leading to social impairment.

The World Health Organization (WHO) recognizes dental caries and periodontal disease, which are the main causes of tooth loss, as a significant public health burden [6, 7]. According to the Global Burden of Disease 2019 [8], in the past three decades, the absolute disease burden of edentulism has nearly doubled even though age-standardized incidence rate, age-standardized prevalence rate, and age-standardized disability-adjusted life year rate of edentulism have declined or stabilized in most countries. As the population ages, the number of older adults and the burden of edentulous disease will continue to increase over the next decade. The proportion is expected to be higher in low-and middle-income countries. In most industrialized countries, oral diseases are the fourth most economically burdening diseases. The total global economic burden of dental disease in 2015 was $544.41 billion, out of which $356.80 billion came from direct treatment spending and $187.61 billion from lost productivity. Of these, 67% of the productivity losses were attributed to severe tooth loss [9].

China is the most populated country in the world. As its aging population increases, the number of patients with dentition loss in China accounts for a more significant proportion of the global total. In recent years, the burden of disease caused by dentition loss has also become a critical component of national health. The prevention and treatment of dentition loss have achieved significant results with the continuous increase in national investment, the constant advancement of medical sciences, and the attention of public health departments. However, according to the data analysis results, the incidence and prevalence of dentition loss in China remain severe. Therefore, long-term systematic research on the disease burden and incidence trend of dentition loss in China is required.

Some studies have shown that the prevalence of age-specific edentulous jaws has decreased in recent years [10] while some studies have reported an increase in the trend [8]. However, previous studies have rarely adjusted for the three distinct effects associated with the trend toward edentulousness, namely age, period, and cohort (APC) effects. Age effects reflect the individual-specific biological and social processes of aging [11]. Menstrual effects are external factors that affect all age groups equally at a given calendar time [12]. Cohort effects reflect the different formative life experiences of successive generations and are an important dimension for understanding how population health status changes over time [13]. Because each temporal dimension uniquely contributes to health research, there is a need to distinguish between these temporal sources of edentulous change. Overall, failure to isolate APC trends may lead to serious biases and provide an incomplete picture of population health trends [13]. Therefore, further study is required on how the incidence and prevalence of edentulous jaws in the Chinese population vary with age, period and cohort. The linkage point regression model, also known as the segmented regression model, is a collection of linear statistical models with the linkage point connecting all models in the collection. This model enables the analysis of the statistical significance of changes over time and thus avoids the lack of an objective point of comparison in the traditional trend analysis based on the linear trend of changes. The linkage point regression model determines whether a statistically significant trend exists in disease rate values in different zones, primarily by comparing the annual percentage change APC and the average annual percentage change AAPC with zero. A confidence interval of the annual percentage change or average annual percentage change containing 0 indicates that the change in the segment is not statistically significant, and vice versa. This present study evaluated the long-term trend of the incidence and prevalence of dentition loss in China from 1990 to 2019 using age-period-cohort (APC) and Joinpoint analysis.

## 2. Methods

### 2.1. Data source

Data were retrieved from the GBD 2019, which provides a comprehensive estimation of incidence, prevalence, death, years of life lost (YLLs), YLDs, and disability-adjusted life years (DALYs) for 369 causes and 87 risk factors or clusters in 204 countries and territories, between 1990 and 2019. These estimates were reported by time, location, age group, and gender [14, 15]. In our study, we retrieved dental data on China from 1990–2019 for trend analysis for each age group (20–24, 25–29, 30–34, 35–39, 40–44, 45–49, 50–54, 55–59, 60–64, 65–69, 70–74, 75–79, 80–84, and 90–94 years of age), prevalence, and YLD of missing teeth, from the official website of the Institute for Health Metrics and Evaluation of GBD 2019 (IHME, http://www.healthdata.org/). The prevalence, incidence, and DALYs of edentulism were estimated using a Bayesian meta-regression model (DisMod-MR 2.1) to ensure consistency between estimates. The complete time series (1990–2019) was recalculated in the GBD to generate comparable values for trend analysis. The primary data supplying the model were derived from a recent systematic review of observational studies on the epidemiology of tooth loss, scientific studies, and health surveys [15]. The disability weight for missing teeth in the GBD 2019 study was 0.067 (0.045–0.095) for estimating DALYs for missing teeth. Age-standardized rates for missing teeth were based on the GBD 2019 global age-standardized population. DALYs are a crucial demographic indicator in the GBD and are the sum of YLLs and YLD. Deaths directly caused by oral disease are uncommon. Therefore, only YLDs were used in this study. YLDs are calculated by summing the frequency (prevalence), severity (weight of disability), and duration of the condition. The reliability of GBD data has been confirmed in the literature [14–17].

### 2.2. Definition of edentulism

The case definition of *edentulism* includes any individual with zero remaining permanent teeth, excluding edentulousness in infancy. This disease is evaluated by quantifying its incidence and estimating the primary sequelae; specifically, asymptomatic and symptomatic edentulism resulting in “great difficulty in eating meat, fruits, and vegetables.” A small body of evidence suggests that edentulism predisposes individuals to an increased risk of ischemic cardiovascular events, including myocardial infarction and stroke. These sparse data have been incorporated into models estimating excess mortality in individuals with complete tooth loss. However, as this association is considered ecological rather than causal, tooth loss was not estimated as an underlying cause of death. Therefore, it was not included in the analysis of risk factors for cardiovascular disease. Missing teeth are defined according to the International Classification of Diseases, Ninth Revision (ICD-9) (Code 155), and ICD-10 (Code C22) [14].

### 2.3. Statistical analysis

Gender-specific incidence, prevalence, and YLD rates were analyzed by Joinpoint regression analysis for the age group of 20–94 years during 1990–2019. In this model, changes in incidence, prevalence, and YLD rates for Chinese men and women across the years were determined by breakpoints to describe temporal trends more visually in the burden of tooth loss. A Monte Carlo permutation method was used to test for significant changes over time. We estimated the APC and the average annual percentage change (AAPC, calculated as geometric weighting from 1990–2019) for each segment identified by the model. The APC and AAPC were used to characterize the changing trends of age-standardized incidence, prevalence, and DALY rates of edentulism; APC/AAPC>0 indicated that the rates increased year by year, and APC/AAPC<0 indicated that the rates decreased year by year during the segment. The National Cancer Institute (NCI) conducted the analysis using the Joinpoint regression program software (version 4.1.0; Statistical Research and Applications Branch, NCI) [15].

APC analysis was performed to determine the relative risk (RR) of tooth loss from age, period, and cohort effects in Chinese men and women, a technique commonly used in epidemiological and sociological fields. The age-specific incidence, prevalence, and YLD rates were recoded into consecutive 5-year periods (1990,1995,2000,2005,2010,and 2015) and 15 successive age groups to fit the model. Based on the equation (period –age = cohort), we classified 20 cohorts of birth (i.e., 1900–1904; 1905–1909; and so forth until 1995–1999). An intrinsic estimator (IE) with a base Poisson log-linear model was used to estimate the APC parameters because of the linear relationship between the three parameters. Additionally, deviance, Akaike’s information criterion (AIC), and Bayesian information criterion (BIC) were applied to test the model’s goodness of fit. Finally, RR (exp[coef.] = e coef.) was used to interpret the estimated parameters of the model. APC analysis was performed using the Stata version 15.0 (Stata Corp, College Station, Texas, United States) [15].

## 3. Results

### 3.1. Secular trends in the incidence, prevalence, and YLD rates of edentulism during 1990–2019

**Figure 1:**
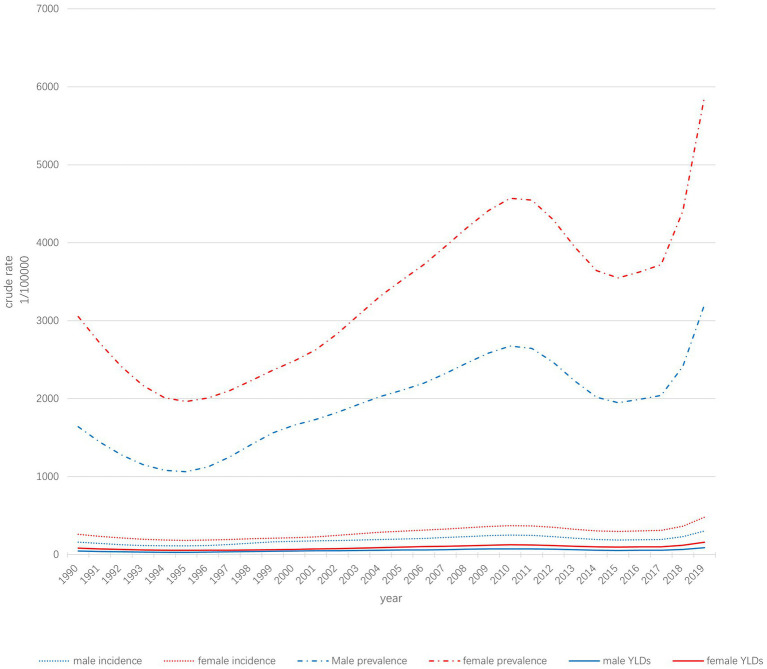
Trends in the incidence, prevalence, and YLDs of dentition loss in the Chinese population.

The trends of the crude incidence, prevalence, and YLD statistics of edentulism in the Chinese population from 1990–2019 are shown in Figures 1. From 1990–2019, the increase in crude incidence in men was 88.14%, from 160.33 to 301.64 per 100,000. The crude incidence rate in women increased from 259.37 to 479.63 per 100,000 (84.92%). The crude prevalence rate increased by 95.41%, from 1640.24 per 100,000 to 3205.16 per 100,000, and by 92.41% from 3051.55 per 100,000 in 1990 to 5871.56 per 100,000, for men and women, respectively. The crude YLD increase in men was 94.12%, from 45.59 per 100,000 to 88.52 per 100,000. In women, the same indicator rose from 83.43 per 100,000 in 1990 to 159.72 per 100,000, a 91.44% increase. Overall, the crude incidence, prevalence, and YLD levels were slightly lower in men than in women; however, these increased more in men than in women.

### 3.2. Secular trends of age-standardized incidence, prevalence, and YLD rates of edentulism during 1990–2019

**Figure 2:**
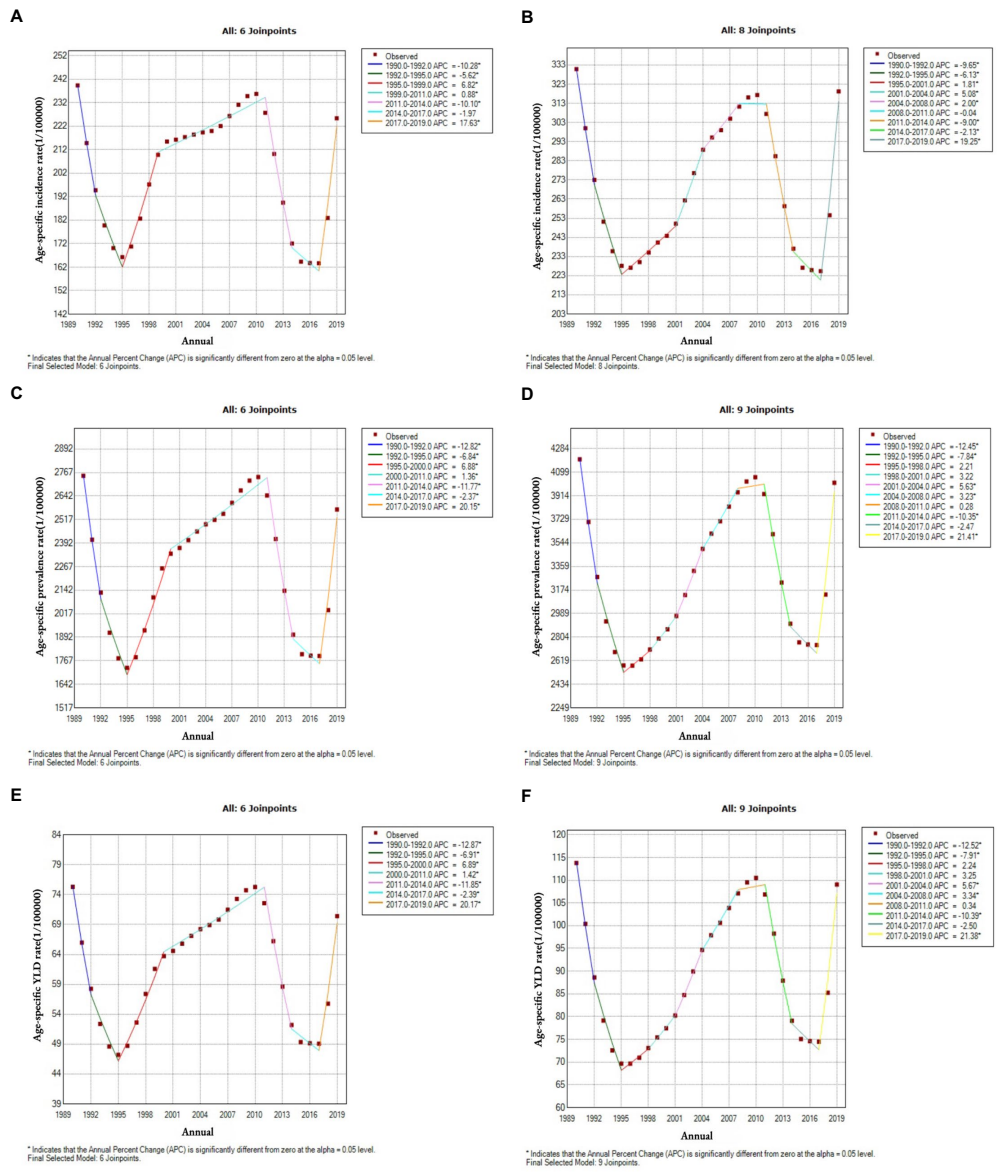
Joinpoint regression analysis of the incidence, prevalence and years lived with disability (YLDs) of edentulism in Chinese men and women (1990–2019). **(A)** incidence in men, **(B)** incidence in women, **(C)** prevalence in men, **(D)** prevalence in women, **(E)** YLDs for men, **(F)** YLDs for women.

The APCs for Chinese men and women of age-standardized incidence, prevalence, and YLD rates are shown in Figures 2. Figure 2A shows the standardized incidence of edentulism in Chinese men from 1990–2019 in seven stages, six of which were significant. It increased by 6.8, 0.9, and 17.6% in 1995–1999, 1999–2011, and 2017–2019, respectively, and decreased by 10.3, 5.6, and 10.1% in 1990–1992, 1992–1995, and 2011–2014, respectively. Over the entire period, the AAPC was −0.3%. Regarding standardized disease rates in women (Figure 2B), the standardized prevalence of edentulism in Chinese women from 1990 to 2019 included nine stages, eight of which were significant: a 1.8, 5.1, 2, and 19.3% increase from 1995–2001, 2001–2004, 2004–2008, and 2017–2019, respectively; the same declined by 9.6, 6.1, 9, and 2.1% in 1990–1992, 1992–1995, 2011–2014, and 2014–2017, respectively. Over the entire period, the AAPC was −0.2%. The overall trends in prevalence and YLD rates resembled the incidence rates (Figures 2C–F).

### 3.3. Estimates of age, period, and cohort effects using the APC model

**Figure 3:**
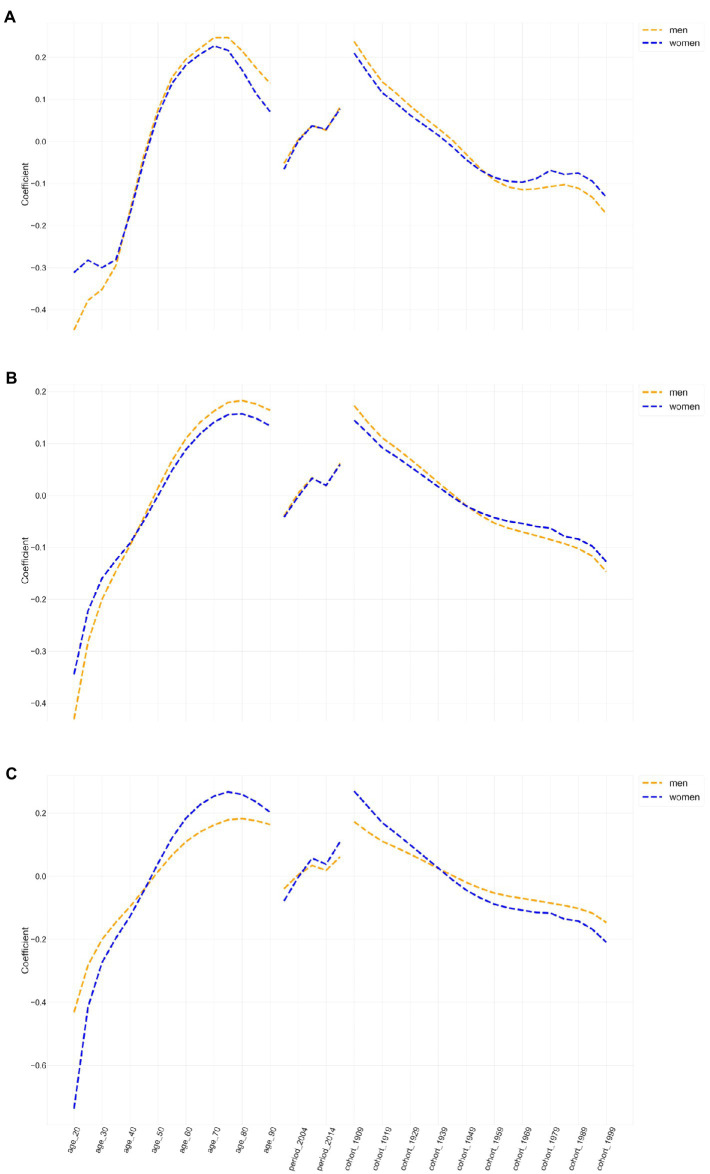
The estimated coefficients of age, period, and cohort effects on incidence, prevalence and years lived with disability rates of edentulism in China (**(A)**: incidence, **(B)**: prevalence, **(C)**: years lived with disability).

Figure 3 shows the estimated coefficients of age, period, and cohort effects for the incidence, prevalence, and YLD rate for edentulism in Chinese men and women. Tables 1–3 show the fit of the APC model, the RR, and the corresponding 95% confidence intervals (95% CI) for the three effects of the incidence, prevalence, and YLD rate of edentulism. The effect coefficients were less than zero in six age groups: 20–24, 25–29, 30–34, 35–39, 40–44, and 45–49 years. After age 50, the age effect coefficient continued to rise, with the age effect for men and women peaking at age 70–74 with RRs of 0.25 (95% CI: 0.23–0.27) and 0.23 (0.20–0.25), respectively. After peaking, the age effect began to decline gradually. The highest prevalence rate was in the 75–79 group, with RRs of 0.18 (0.17–0.19) and 0.16 (0.14–0.17) for men and women, respectively. The highest YLD rate was in the 75–79 group, with RRs of 0.18 (0.17–0.19) and 0.27 (0.25–0.28) for men and women, respectively.

**Table 1:** Relative risk and 95% CI for the prevalence of edentulousness by age, period, and cohort in the Chinese population.

Factors	Men				Women			
	Effect coefficient	standard error	*Z* value	*P* value	Effect coefficient	standard error	*Z* value	*p* value
age_20	−0.43 (−0.45to-0.41)	0.01	−37.64	<0.0001	−0.34 (−0.37to-0.32)	0.01	−33.11	<0.0001
age_25	−0.28 (−0.30to-0.26)	0.01	−30.66	<0.0001	−0.22 (−0.24to-0.21)	0.01	−25.98	<0.0001
age_30	−0.20 (−0.22to-0.18)	0.01	−23.39	<0.0001	−0.16 (−0.18to-0.14)	0.01	−19.78	<0.0001
age_35	−0.15 (−0.16to-0.13)	0.01	−17.76	<0.0001	−0.12 (−0.14to-0.11)	0.01	−15.84	<0.0001
age_40	−0.10 (−0.11to-0.08)	0.01	−12.22	<0.0001	−0.09 (−0.11to-0.08)	0.01	−11.94	<0.0001
age_45	−0.04 (−0.06to-0.03)	0.01	−5.51	<0.0001	−0.05 (−0.06to-0.03)	0.01	−6.49	<0.0001
age_50	0.01 (0.00to0.03)	0.01	2.05	0.04	0.00 (−0.01to0.01)	0.01	−0.04	0.97
age_55	0.07 (0.05to0.08)	0.01	9.82	<0.0001	0.05 (0.03to0.06)	0.01	7.03	<0.0001
age_60	0.11 (0.10to0.12)	0.01	17.20	<0.0001	0.09 (0.08to0.10)	0.01	13.67	<0.0001
age_65	0.14 (0.13to0.15)	0.01	23.55	<0.0001	0.12 (0.11to0.13)	0.01	19.42	<0.0001
age_70	0.16 (0.15to0.17)	0.01	28.92	<0.0001	0.14 (0.13to0.15)	0.01	24.49	<0.0001
age_75	0.18 (0.17to0.19)	0.01	33.41	<0.0001	0.16 (0.14to0.17)	0.01	28.20	<0.0001
age_80	0.18 (0.17to0.19)	0.01	34.79	<0.0001	0.16 (0.15to0.17)	0.01	29.01	<0.0001
age_85	0.18 (0.17to0.19)	0.01	32.98	<0.0001	0.15 (0.14to0.16)	0.01	27.06	<0.0001
age_90	0.16 (0.15to0.18)	0.01	27.87	<0.0001	0.13 (0.12to0.15)	0.01	22.19	<0.0001
period_1994	−0.08 (−0.09to-0.07)	0.00	−19.98	<0.0001	−0.07 (−0.08to-0.06)	0.00	−17.18	<0.0001
period_1999	−0.04 (−0.05to-0.03)	0.00	−10.68	<0.0001	−0.04 (−0.05to-0.03)	0.00	−11.15	<0.0001
period_2004	0.00 (0.00to0.01)	0.00	1.18	0.24	0.00 (−0.01to0.00)	0.00	−0.61	0.54
period_2009	0.03 (0.03to0.04)	0.00	9.53	<0.0001	0.03 (0.03to0.04)	0.00	9.13	<0.0001
period_2014	0.02 (0.01to0.03)	0.00	5.12	<0.0001	0.02 (0.01to0.03)	0.00	5.26	<0.0001
period_2019	0.06 (0.05to0.07)	0.00	16.85	<0.0001	0.06 (0.05to0.07)	0.00	16.21	<0.0001
cohort_1904	0.21 (0.18to0.23)	0.01	16.78	<0.0001	0.18 (0.15to0.20)	0.01	13.88	<0.0001
cohort_1909	0.17 (0.16to0.19)	0.01	19.65	<0.0001	0.14 (0.13to0.16)	0.01	15.87	<0.0001
cohort_1914	0.14 (0.13 to0.15)	0.01	19.26	<0.0001	0.12 (0.10to0.13)	0.01	15.84	<0.0001
cohort_1919	0.11 (0.10to0.12)	0.01	17.43	<0.0001	0.09 (0.08to0.10)	0.01	13.94	<0.0001
cohort_1924	0.09 (0.08to0.10)	0.01	15.69	<0.0001	0.07 (0.06to0.09)	0.01	12.39	<0.0001
cohort_1929	0.07 (0.06to0.08)	0.01	12.89	<0.0001	0.06 (0.04to0.07)	0.01	9.90	<0.0001
cohort_1934	0.05 (0.04to0.06)	0.01	8.19	<0.0001	0.04 (0.02to0.05)	0.01	6.05	<0.0001
cohort_1939	0.02 (0.01to0.04)	0.01	3.97	<0.0001	0.02 (0.00to0.03)	0.01	2.61	0.01
cohort_1944	0.00 (−0.01to0.02)	0.01	0.40	0.69	0.00 (−0.02to0.01)	0.01	−0.38	0.71
cohort_1949	−0.02 (−0.03to-0.01)	0.01	−2.70	0.01	−0.02 (−0.03to-0.01)	0.01	−2.80	0.01
cohort_1954	−0.04 (−0.05to-0.02)	0.01	−4.99	<0.0001	−0.03 (−0.05to-0.02)	0.01	−4.35	<0.0001
cohort_1959	−0.05 (−0.07to-0.04)	0.01	−6.66	<0.0001	−0.04 (−0.06to-0.03)	0.01	−5.44	<0.0001
cohort_1964	−0.06 (−0.08to-0.05)	0.01	−7.64	<0.0001	−0.05 (−0.07to-0.03)	0.01	−6.20	<0.0001
cohort_1969	−0.07 (−0.09to-0.05)	0.01	−8.36	<0.0001	−0.05 (−0.07to-0.04)	0.01	−6.67	<0.0001
cohort_1974	−0.08 (−0.09 to-0.06)	0.01	−9.01	<0.0001	−0.06 (−0.08to-0.04)	0.01	−7.31	<0.0001
cohort_1979	−0.09 (−0.10to-0.07)	0.01	−9.02	<0.0001	−0.06 (−0.08to-0.05)	0.01	−7.09	<0.0001
cohort_1984	−0.09 (−0.11to-0.07)	0.01	−8.75	<0.0001	−0.08 (−0.10to-0.06)	0.01	−7.91	<0.0001
cohort_1989	−0.10 (−0.13to-0.08)	0.01	−8.18	<0.0001	−0.08 (−0.11to-0.06)	0.01	−7.26	<0.0001
cohort_1994	−0.12 (−0.15to-0.09)	0.02	−7.29	<0.0001	−0.10 (−0.13to-0.07)	0.01	−6.65	<0.0001
cohort_1999	−0.15 (−0.20to-0.09)	0.03	−5.31	<0.0001	−0.13 (−0.18to-0.08)	0.02	−5.12	<0.0001
intercept	1.22 (1.22to1.23)	0.00	496.11	<0.0001	1.29 (1.29to1.30)	0.00	545.39	<0.0001
Deviance	0.15			0.17				
AIC	−2.70			−2.57				
BIC	−233.84			−233.82				

Akaike Information Criterion (AIC); Bayesian Information Criterion (BIC).

**Table 2:** Relative risk and 95% CI for the incidence of edentulousness by age, period, and cohort in the Chinese population.

Factors	Men				Women			
	Effect coefficient	standard error	*Z* value	*p* value	Effect coefficient	standard error	*Z* value	*p* value
age_20	−0.45 (−0.50to-0.40)	0.03	−16.86	0.00	−0.31 (−0.36to-0.26)	0.02	−12.52	0.00
age_25	−0.38 (−0.42to-0.33)	0.02	−16.54	0.00	−0.28 (−0.33to-0.24)	0.02	−12.77	0.00
age_30	−0.35 (−0.40to-0.31)	0.02	−15.76	0.00	−0.30 (t-0.34o-0.26)	0.02	−13.42	0.00
age_35	−0.29 (−0.34to-0.25)	0.02	−13.78	0.00	−0.28 (−0.32to-0.24)	0.02	−12.65	0.00
age_40	−0.16 (−0.20to-0.13)	0.02	−8.52	0.00	−0.17 (−0.21 to-0.13)	0.02	−8.41	0.00
age_45	−0.03 (−0.07to0.00)	0.02	−1.87	0.06	−0.04 (−0.08to-0.01)	0.02	−2.36	0.02
age_50	0.08 (0.04to0.11)	0.02	4.74	0.00	0.06 (0.03to0.10)	0.02	3.70	0.00
age_55	0.15 (0.12to0.18)	0.01	10.35	0.00	0.14 (0.11to0.17)	0.02	8.60	0.00
age_60	0.20 (0.17to0.22)	0.01	14.41	0.00	0.18 (0.15to0.21)	0.01	12.17	0.00
age_65	0.22 (0.20to0.25)	0.01	17.65	0.00	0.21 (0.18to0.23)	0.01	14.88	0.00
age_70	0.25 (0.22to0.27)	0.01	21.24	0.00	0.23 (0.20to0.25)	0.01	17.44	0.00
age_75	0.25 (0.23to0.27)	0.01	22.22	0.00	0.22 (0.19to0.24)	0.01	17.23	0.00
age_80	0.22 (0.19to0.24)	0.01	19.29	0.00	0.17 (0.15 to0.20)	0.01	13.35	0.00
age_85	0.18 (0.15to0.20)	0.01	15.01	0.00	0.11 (0.09to0.14)	0.01	8.53	0.00
age_90	0.14 (0.11to0.16)	0.01	10.52	0.00	0.07 (0.04to0.10)	0.01	4.74	0.00
period_1994	−0.10 (−0.12to-0.08)	0.01	−11.15	0.00	−0.08 (−0.10to-0.06)	0.01	−8.20	0.00
period_1999	−0.05 (−0.07to-0.04)	0.01	−6.43	0.00	−0.07 (−0.08to-0.05)	0.01	−7.26	0.00
period_2004	0.00 (−0.01to0.02)	0.01	0.53	0.59	0.00 (−0.02to0.02)	0.01	0.08	0.94
period_2009	0.04 (0.02to0.05)	0.01	4.88	0.00	0.04 (0.02to0.05)	0.01	4.31	0.00
period_2014	0.03 (0.01to0.04)	0.01	3.34	0.00	0.03 (0.01to0.05)	0.01	3.27	0.00
period_2019	0.08 (0.07to0.10)	0.01	10.13	0.00	0.08 (0.06to0.09)	0.01	8.77	0.00
cohort_1904	0.29 (0.24to0.34)	0.03	10.82	0.00	0.24 (0.18to0.30)	0.03	7.84	0.00
cohort_1909	0.24 (0.20to0.28)	0.02	12.35	0.00	0.21 (0.17to0.25)	0.02	9.47	0.00
cohort_1914	0.19 (0.16to0.22)	0.02	11.88	0.00	0.16 (0.13to0.20)	0.02	8.91	0.00
cohort_1919	0.14 (0.12to0.17)	0.01	10.37	0.00	0.12 (0.09to0.15)	0.02	7.37	0.00
cohort_1924	0.11 (0.09to0.14)	0.01	9.26	0.00	0.09 (0.06to0.12)	0.01	6.40	0.00
cohort_1929	0.08 (0.06to0.11)	0.01	7.35	0.00	0.06 (0.04to0.09)	0.01	4.76	0.00
cohort_1934	0.06 (0.03to0.08)	0.01	4.64	0.00	0.04 (0.01to0.07)	0.01	2.80	0.01
cohort_1939	0.03 (0.01to0.06)	0.01	2.35	0.02	0.01 (−0.01to0.04)	0.01	1.02	0.31
cohort_1944	0.00 (−0.02to0.03)	0.14	0.25	0.81	−0.01 (−0.04to0.02)	0.02	−0.78	0.44
cohort_1949	−0.03 (−0.06to0.00)	0.02	−1.95	0.05	−0.04 (−0.08to0.01)	0.02	−2.54	0.01
cohort_1954	−0.06 (−0.10to-0.03)	0.02	−3.78	0.00	−0.07 (−0.10to-0.03)	0.02	−3.73	0.00
cohort_1959	−0.09 (−0.13to-0.06)	0.02	−5.02	0.00	−0.09 (−0.12to-0.05)	0.02	−4.38	0.00
cohort_1964	−0.11 (−0.15to-0.07)	0.02	−5.55	0.00	−0.09 (−0.13to-0.05)	0.02	−4.61	0.00
cohort_1969	−0.11 (−0.15to-0.07)	0.02	−5.61	0.00	−0.10 (−0.14to-0.06)	0.02	−4.58	0.00
cohort_1974	−0.11 (−0.15to-0.07)	0.02	−5.33	0.00	−0.09 (−0.13to-0.05)	0.02	−4.12	0.00
cohort_1979	−0.11 (−0.15to-0.06)	0.02	−4.58	0.00	−0.07 (−0.11to-0.02)	0.02	−2.95	0.00
cohort_1984	−0.10 (−0.15to-0.05)	0.03	−3.91	0.00	−0.08 (−0.13to-0.03)	0.03	−3.02	0.00
cohort_1989	−0.11 (−0.17to-0.05)	0.03	−3.65	0.00	−0.07 (−0.13to-0.02)	0.03	−2.57	0.01
cohort_1994	−0.13 (−0.21to-0.06)	0.04	−3.49	0.00	−0.09 (−0.16to-0.02)	0.04	−2.65	0.01
cohort_1999	−0.17 (−0.30to-0.05)	0.06	−2.74	0.01	−0.13 (−0.25 to-0.02)	0.06	−2.30	0.02
intercept	0.86 (0.85to0.87)	0.01	151.70	0.00	0.94 (0.92to0.95)	0.01	162.60	0.00
Deviance	0.36				0.49			
AIC	−1.84				−1.53			
BIC	−233.63				−233.50			

Akaike Information Criterion (AIC); Bayesian Information Criterion (BIC).

**Table 3:** Relative risk and 95% CI for the years lived with disability of edentulousness by age, period, and cohort in the Chinese population.

Factors	Men				Women			
	Effect coefficient	standard error	*Z* value	*p* value	Effect coefficient	standard error	*Z* value	*p* value
age_20	−0.43 (−0.45to-0.41)	0.01	−37.64	0.00	−0.74 (−0.79to-0.68)	0.03	−25.55	0.00
age_25	−0.28 (−0.30to-0.26)	0.01	−30.66	0.00	−0.41 (−0.45to-0.38)	0.02	−20.91	0.00
age_30	−0.20 (−0.22to-0.18)	0.01	−23.39	0.00	−0.27 (−0.31to-0.24)	0.02	−15.57	0.00
age_35	−0.15 (−0.16to-0.13)	0.01	−17.76	0.00	−0.20 (−0.23to-0.16)	0.02	−11.86	0.00
age_40	−0.10 (−0.11to-0.08)	0.01	−12.22	0.00	−0.13 (−0.16to-0.10)	0.02	−8.17	0.00
age_45	−0.04 (−0.06to-0.03)	0.01	−5.51	0.00	−0.05 (−0.07to-0.02)	0.01	−3.12	0.00
age_50	0.01 (0.00to0.03)	0.01	2.05	0.04	0.04 (0.01to0.07)	0.01	3.04	0.00
age_55	0.07 (0.05to0.08)	0.01	9.82	0.00	0.12 (0.10to0.15)	0.01	9.83	0.00
age_60	0.11 (0.10to0.12)	0.01	17.20	0.00	0.18 (0.16to0.21)	0.01	16.31	0.00
age_65	0.14 (0.13to0.15)	0.01	23.55	0.00	0.23 (0.21to0.25)	0.01	21.97	0.00
age_70	0.16 (0.15to0.17)	0.01	28.92	0.00	0.25 (0.24 to0.27)	0.01	26.74	0.00
age_75	0.18 (0.17to0.19)	0.01	33.41	0.00	0.27 (0.25to0.28)	0.01	29.84	0.00
age_80	0.18 (0.17to0.19)	0.01	34.79	0.00	0.26 (0.24to0.28)	0.01	29.44	0.00
age_85	0.18 (0.17to0.19)	0.01	32.98	0.00	0.24 (0.22to0.25)	0.01	25.92	0.00
age_90	0.16 (0.15to0.18)	0.01	27.87	0.00	0.20 (0.18to0.22)	0.01	19.89	0.00
period_1994	−0.08 (−0.09to-0.07)	0.00	−19.98	0.00	−0.12 (−0.14to-0.11)	0.01	−16.92	0.00
period_1999	−0.04 (−0.05to-0.03)	0.00	−10.68	0.00	−0.08 (−0.09to-0.07)	0.01	−11.78	0.00
period_2004	0.00 (0.00to0.01)	0.00	1.18	0.24	0.00 (−0.02to0.01)	0.01	−0.61	0.54
period_2009	0.03 (0.03to0.04)	0.00	9.53	0.00	0.06 (0.05to0.07)	0.01	9.30	0.00
period_2014	0.02 (0.01to0.03)	0.00	5.12	0.00	0.04 (0.03to0.05)	0.01	5.88	0.00
period_2019	0.06 (0.05to0.07)	0.00	16.85	0.00	0.11 (0.10to0.12)	0.01	17.19	0.00
cohort_1904	0.21(0.18to0.23)	0.01	16.78	0.00	0.32 (0.28to0.37)	0.02	15.59	0.00
cohort_1909	0.17 (0.16to0.19)	0.01	19.65	0.00	0.27 (0.24to0.30)	0.02	17.80	0.00
cohort_1914	0.14 (0.13 to0.15)	0.01	19.26	0.00	0.22 (0.20to0.24)	0.01	17.67	0.00
cohort_1919	0.11 (0.10to0.12)	0.01	17.43	0.00	0.17 (0.15to0.19)	0.01	15.69	0.00
cohort_1924	0.09 (0.08to0.10)	0.01	15.69	0.00	0.14 (0.12to0.16)	0.01	13.72	0.00
cohort_1929	0.07 (0.06to0.08)	0.01	12.89	0.00	0.10 (0.08to0.12)	0.01	10.75	0.00
cohort_1934	0.05 (0.04to0.06)	0.01	8.19	0.00	0.06 (0.04to0.08)	0.01	6.26	0.00
cohort_1939	0.02 (0.01to0.04)	0.01	3.97	0.00	0.03 (0.00to0.05)	0.01	2.39	0.02
cohort_1944	0.00 (−0.01to0.02)	0.01	0.40	0.69	−0.01 (−0.03to0.01)	0.01	−0.92	0.36
cohort_1949	−0.02 (−0.03to-0.01)	0.01	−2.70	0.01	−0.04 (−0.07to-0.02)	0.01	−3.35	0.00
cohort_1954	−0.04 (−0.05to-0.02)	0.01	−4.99	0.00	−0.07 (−0.10to-0.04)	0.01	−4.85	0.00
cohort_1959	−0.05 (−0.07 to-0.04)	0.01	−6.66	0.00	−0.09 (−0.12to-0.06)	0.02	−5.83	0.00
cohort_1964	−0.06 (−0.08 to-0.05)	0.01	−7.64	0.00	−0.10 (−0.13to-0.07)	0.02	−6.26	0.00
cohort_1969	−0.07 (−0.09to-0.05)	0.01	−8.36	0.00	−0.11 (−0.14to-0.07)	0.02	−6.41	0.00
cohort_1974	−0.08 (−0.09 to-0.06)	0.01	−9.01	0.00	−0.12 (−0.15to-0.08)	0.02	−6.50	0.00
cohort_1979	−0.09 (−0.10to-0.07)	0.01	−9.02	0.00	−0.12 (−0.15to-0.08)	0.02	−5.98	0.00
cohort_1984	−0.09 (−0.11to-0.07)	0.01	−8.75	0.00	−0.14 (−0.18to-0.09)	0.02	−6.11	0.00
cohort_1989	−0.10 (−0.13to-0.08)	0.01	−8.18	0.00	−0.14 (−0.19to-0.09)	0.03	−5.34	0.00
cohort_1994	−0.12 (−0.15to-0.09)	0.02	−7.29	0.00	−0.17 (−0.24to-0.10)	0.04	−4.67	0.00
cohort_1999	−0.15 (−0.20to-0.09)	0.03	−5.31	0.00	−0.21 (−0.34to-0.07)	0.07	−3.02	0.00
intercept	1.22 (1.22to1.23)	0.00	496.11	0.00	0.71 (0.70 to0.72)	0.01	137.18	0.00
Deviance	0.15				0.18			
AIC	−2.70				−2.54			
BIC	−233.84				−233.81			

Akaike Information Criterion (AIC); Bayesian Information Criterion (BIC).

The estimated period effect showed a progressive increase in the risk of YLD prevalence among Chinese men and women, suggesting that the period effect contributed significantly to the increased burden of YLD. From 1990–2019, the incidence risk shifted from negative to positive in Chinese men and women, increasing by 0.18 and 0.16, with an increased prevalence risk of 0.14 and 0.13, respectively, and an increased YLD risk of 0.14 and 0.23, respectively.

The cohort effect showed a decreasing trend for the risk of developing dental agenesis in the Chinese population during the observation period 1990–2019. The net cohort effect decreased gradually with birth cohorts of later generations. The 1904–1908 cohort experienced a higher risk of dental agenesis than later birth cohorts. The lowest cohort effect for the risk of developing dental agenesis was found in the 1999–2003 birth cohort. The negative estimated coefficients for these cohorts showed weak effects. The downward trend in cohort effects in incidence, prevalence, and DALYs was relatively consistent for Chinese men and women.

## 4. Discussion

This study examined trends in the burden of dental agenesis in China over the past 30 years. To the best of our knowledge, this is the first study on the epidemiological trends of dental agenesis in China that used linkage point analysis and APC models. Our results showed that the crude incidence, prevalence, and YLDs of edentulism in our population continuously increased while the age-standardized rates decreased. This opposite trend in crude and age-standardized rates was mainly due to the progression of aging, as an increase in the proportion of older adults in the population would cause an increase in the crude incidence, prevalence, and DALY rates of edentulism. According to the literature, in 2017, the global population aged 60 and older reached 962 million, more than twice the 382 million older adults in 1980. By 2050, the number of older adults is expected to double again to a projected 2.1 billion. Some studies predict that by 2030, the proportion of the older adult population (65 years and older) in China will reach 19.25% [18]. Thus, tooth loss would remain a substantial economic burden for China.

Comparing the data of this study from the GBD with that of the national epidemiological surveys, we found that the second national oral health survey in 1995 showed that the prevalence of dental agenesis in those 65–74 years old was 10.51%, while the same was 10.04% in the same year based on the estimation in the GBD 2019. The third Chinese National Oral Health Epidemiology Survey (CNOHES) [19] showed that the prevalence of dental agenesis in 65–74-year-olds was 6.8%. In comparison, the GBD projected it as 15.7% in 2005 based on the estimation of the GBD 2019. The prevalence of missing teeth in China was 4.5% in the Fourth CNOHES of 65–74-year-olds in 2015 [20, 21], and the GBD 2019 estimates showed it as 9.6% for the same year. Although there are some differences between the absolute values of the data of the two studies, their trends are consistent. The differences between the prevalence estimates of the GBD 2019 and the CNOHES may be caused by the diversity of underlying data sources, the survey population’s age and sex composition, and the setting of modeling estimation parameters. According to the Joinpoint trend analysis from 1995 to 2015, the years 1995–2005 showed an increasing trend, and the years 2005–2015 experienced an increasing and then decreasing trend, with the year 2011 as the boundary. The Joinpoint trend analysis makes the prevalence trend more accurate than the decennial oral epidemiological survey does. In the years 2011–2015, the decrease in prevalence may be attributed to the Chinese Dental Association’s launch and implementation of the “healthy mouth, happy family; care for your children, prevent dental caries” campaign on Love Teeth Day for three consecutive years from 2011–2013. The series of activities in the campaign may have increased the general public’s awareness of dental care. Additionally, the rapid development of China’s economy has improved people’s quality of life.

APC analysis showed that when period and cohort effects were controlled, the incidence, prevalence, and risk of YLDs for edentulism in the Chinese population increased with age. However, the increase was limited in nature. For both men and women, the standardized incidence peaked for those aged 70–74 years, and prevalence and YLDs peaked for those aged 75–79 years, after which the growth slowed. In our study, the age effect trend in China is consistent with the report of the Fourth CNOHES in China [20, 21]. Aging is a significant risk factor for tooth loss. Missing teeth are common in the older adult population [22]. There is a complex and nonlinear association between tooth loss and age, and these similarities between the GBD results and previous research investigations enhance the validity and reliability of the latter. Previous studies have shown that age and reduced access to dental care due to physical and cognitive impairment can directly affect periodontal health through the immune system and cellular aging [6]. More evidence confirms that people with untreated or not optimally controlled diabetes are at higher risk for periodontal disease, which would accelerate tooth loss [23]. Many studies have shown that the repair mechanisms of dental tissues in older adults are weak due to aging. Therefore, any physiological changes, such as aging, external impact (e.g., oral impact in an accident), or dental disease, can eventually lead to tooth loss. In recent years, due to advances in treatment options, young people are at less risk of tooth loss than older people.

The incidence, prevalence, and disability-adjusted annual rate of edentulism showed a gradual increase throughout the period in China. This trend suggests that period effects are critical in the increase of the edentulism disease burden. According to the results of our study, people’s oral condition has improved considerably in the past few decades due to the improvement of medical technology, the increase in the number of dentists and dental institutions, and people’s heightened health awareness and other favorable conditions. However, the risk of tooth loss has increased over time with the increase in the pressure in people’s lives and work; the change in dietary habits, such as the increase in the intake of sugar, tobacco [24, 25], and alcohol [26, 27]; and the increase in the standardized incidence of chronic non-communicable diseases, such as caries, periodontal disease, diabetes, hypertension, and metabolic diseases. Some studies have shown that significant causes of tooth loss are untreated dental caries and periodontal disease. According to the National Institute of Dental and Craniofacial Research, periodontal disease is one of the most common risk factors for tooth loss. A study by Shammari et al. indicated that severe periodontitis is the leading cause of tooth extraction, affecting 5–15% of the population in many countries [28]. Furthermore, dental caries lead to tooth extraction. Anand examined records of 1,791 permanent teeth extracted in the dental clinic of the Indian Dental Institute and found that dental caries accounted for 39.5% of the extractions. Decay was a significant cause of tooth loss [29]. Once oral disease occurs, timely and proper oral treatment is the key to stopping its deterioration. Otherwise, lack of effective treatment will lead to tooth loss. Therefore, while strengthening the prevention and treatment of tooth loss, we must also focus on caries, periodontal disease, diabetes, and hypertension as the high-risk groups of tooth loss. Different strategies are adopted to achieve the goal of oral health. Since dental caries and periodontal disease are preventable and controllable, increasing health insurance coverage for preventive care and conservative treatment of oral diseases, especially periodontal disease, will significantly reduce the disease burden associated with tooth loss. At the same time, it will promote healthy lifestyles to meet public health challenges. Reducing oral health inequities will also help reduce tooth loss and thus reduce the disease burden [30].

The net cohort effect reflects changes in early life circumstances, as exposure to certain adverse environmental, socioeconomic, and behavioral factors at young ages may have adverse effects later in life [15, 31]. We found a monotonic decreasing trend in the cohort effect of standardized incidence, prevalence, and the YLD rate of edentulism in our population. Thus, those born in the early birth cohort had a higher risk of edentulism than those born in the later birth cohort. This outcome is due to the higher extraction rate caused by the harsh economic conditions and living environment of the early birth cohort and the low level of oral health care. The later birth cohort benefited from preventive measures and advanced restorative dentistry, leading to a reduced rate of tooth loss. Conversely, the downward trend in cohort effects may be caused by economic development and environmental and cultural improvements. An unhealthy environment, poor oral health care, and low socioeconomic status influence intrauterine and early childhood development. They may also have profound adverse effects on oral health status, contributing to an increased risk of tooth loss in adulthood [15, 32, 33]. Investigating historical background is essential when determining the incidence of edentulism in different populations. With the development of the economy and health care system, later generations living in a better childhood environment have had better nutrition and awareness of information related to oral diseases, and these can play crucial roles in reducing the risk of tooth loss [15].

Our study found that the incidence, prevalence, and YLDs of edentulism were higher in women than in men in the Chinese population over the past 30 years; however, this study was based on estimated values, and no secondary statistical analysis could be performed. In addition, some studies showed that although the prevalence was higher in women, it was not statistically significant [34]. For example, according to the Third CNOHES [19], the prevalence was higher in women in the 65–74 age group, while the Fourth CNOHES [21] contradicted this finding. Therefore, more studies are needed to confirm whether a difference exists between the prevalence of edentulism in men and women.

Another interesting finding is the following two inconsistencies in the APC model analysis. First, using the 40–45-year-old group as the boundary, we found that the age effect of standardized incidence and prevalence is higher in women under 40 than in men. The age effect of YLDs is lower in women than in men, and the situation is reversed for those above 45 years. This finding suggests that younger women with missing teeth are more inclined to restoration. In comparison, older women have lower restoration rates than men, and the above findings are consistent with those of the Fourth CNOHES [21]. Such trends may be related to young and middle-aged Chinese consumption habits and beliefs.

Second, the cohort effect of incidence and prevalence was higher in women born after 1949 than in men, while the cohort effect of YLD was lower in women than in men. This finding suggests that women born after 1949 are more inclined than men to restore missing teeth, concluding that the high-risk group is middle-aged and older women, which should be analyzed in terms of systemic factors such as estrogen. In conclusion, older women comprise the group with the highest burden of tooth loss. Therefore, we should take measures to prevent tooth loss and increase patients’ restoration rate to improve the quality of life of the older adult population.

This study had some limitations. First, the data presented in our study were obtained from the GBD 2019. Some values were estimated rather than directly measured; thus, they may be inaccurate. However, several methods were used to reduce bias, including misclassification corrections and redistribution of garbage codes. The literature and IHME annual reports have confirmed the reliability of this source [17, 30]. The GBD 2019 study is a valuable addition to the CNOHES. It could help assess long-term trends in the burden of dental disease, considering the paucity of recorded data showing such long-term trends [35]. Second, we excluded the 0–20 and 95+ age groups. The disease burden due to missing teeth in the 0–20 age group is negligible. However, although the ≥95 age group was recorded as a cohort in the GBD 2019, it does not fit the fixed format of the APC model. Finally, the interpretation of the results is focused on the population level rather than the individual level, rendering it challenging to avoid ecological fallacy; thus, more individual-based studies are needed to confirm the findings of the current study. Finally, because China has a huge geographical region with various ethnic groups, the amount of data available to assess the differences in frequency among different provinces and ethnic groups in China is limited.

In the future, if such data on the incidence and prevalence of edentulism at the provincial and even county and municipal levels in China is obtained, classical spatial statistical analysis such as global and local autocorrelation and high-low clustering can be applied to study the spatial aggregation of edentulism. Accordingly, we can combine the age-period-effect model to construct a spatiotemporal data model to study the patterns of edentulism over time and space at the same time.

## 5. Conclusion

From 1990 to 2019, overall crude incidence, prevalence, and YLD rates in China showed an increasing trend, while age-standardized incidence, prevalence, and YLD rates showed a slightly decreasing trend. The incidence, prevalence, and YLDs of edentulism were higher in women than in men in the Chinese population over the past 30 years. However, more studies are needed to confirm whether they differ directly. The age effect showed a definite upward trend in younger age groups, and the population aged 60–75 years was at high risk of developing edentulism. The period effect may be a more critical factor in the increased burden of tooth loss. Therefore, there is an urgent need for timely primary intervention and increased global awareness of oral health, especially in high-risk groups, such as middle-aged and older women.

## Data availability statement

The original contributions presented in the study are included in the article/Supplementary material, further inquiries can be directed to the corresponding authors.

## Author contributions

XS, XQ, and XZ made substantial contributions to the conception and design of the study. XQ, HH, and JH contributed to the acquisition, analysis, and interpretation of data. XQ, XY, XS, JH, and HH contributed to drafting the article and revising the content critically. XZ and XS reviewed the manuscript. All authors contributed to the article and approved the submitted version.

## Funding

This study was funded by the National Natural Science Foundation of China as part of the Regional Science Foundation Program (grant number: 82060202).

## Conflict of interest

The authors declare that the research was conducted in the absence of any commercial or financial relationships that could be construed as a potential conflict of interest.

## Publisher’s note

All claims expressed in this article are solely those of the authors and do not necessarily represent those of their affiliated organizations, or those of the publisher, the editors and the reviewers. Any product that may be evaluated in this article, or claim that may be made by its manufacturer, is not guaranteed or endorsed by the publisher.
